# Association Between Body Mass Index and Clinical Outcomes of CDK4/6 Inhibitors in HR+/HER2− Metastatic Breast Cancer: A Real-World Cohort Study

**DOI:** 10.3390/jcm15041671

**Published:** 2026-02-23

**Authors:** Seval Orman, Miray Aydoğan, Nisanur Sarıyar Busery, Sedat Yıldırım, Hacer Şahika Yıldız, Hamit Bal, Utku Dönem Gündoğdu, Seval Ay Ersoy, Deniz Işık, Hatice Odabaş, Nedim Turan

**Affiliations:** Department of Medical Oncology, Health Science University, Kartal Dr Lutfi Kırdar City Hospital, Istanbul 34865, Türkiye; mirayaydogan1991@gmail.com (M.A.); sariyarnisanur@gmail.com (N.S.B.); rezansedat@hotmail.com (S.Y.); h.sahikayildiz@gmail.com (H.Ş.Y.); hamitbal@yahoo.com (H.B.); drutkudonem@gmail.com (U.D.G.); drsevalay@gmail.com (S.A.E.); dnz.1984@yahoo.com (D.I.); odabashatice@yahoo.com (H.O.); turan.nedim@hotmail.com (N.T.)

**Keywords:** body mass index, CDK4/6 inhibitors, metastatic breast cancer, propensity score matching, real-world data

## Abstract

**Background:** Body mass index (BMI) has been widely investigated as a potential prognostic factor in breast cancer; however, its clinical relevance in patients with hormone receptor-positive/human epidermal growth factor receptor 2-negative (HR+/HER2−) metastatic breast cancer treated with CDK4/6 inhibitors remains controversial, particularly in contemporary real-world settings. This study aimed to evaluate the association between baseline BMI and clinical outcomes, including survival and treatment-related toxicity, in a real-world cohort. **Methods:** This single-centre retrospective observational cohort study included patients with HR+/HER2− metastatic breast cancer treated with endocrine therapy and a CDK4/6 inhibitor (palbociclib or ribociclib) in the metastatic setting between January 2018 and May 2025. Patients were categorised by baseline BMI (<25 vs. ≥25 kg/m^2^). Progression-free survival (PFS) and overall survival (OS) were assessed using the Kaplan–Meier method and Cox proportional hazards models. To minimise confounding, propensity score matching (PSM) with a 1:3 nearest-neighbour algorithm was performed. Non-linear associations between continuous BMI and survival outcomes were explored using restricted cubic spline analyses. Treatment-related adverse events were evaluated according to CTCAE v5.0. **Results:** A total of 456 patients were included; 321 (70.4%) had a BMI ≥ 25 kg/m^2^, and 135 (29.6%) had a BMI < 25 kg/m^2^. Propensity score matching produced a balanced cohort of 220 patients. The reduction in sample size after matching reflects the need to achieve close baseline comparability between groups. In the matched cohort, no statistically significant differences in PFS (log-rank *p* = 0.55) or OS (log-rank *p* = 0.31) were observed across BMI categories. BMI was not an independent predictor of PFS or OS in multivariable analyses. However, restricted cubic spline modelling revealed a non-linear relationship between continuous BMI and survival outcomes, with increased risk at extreme BMI values, underscoring the limitations of dichotomous BMI categorisation. **Conclusions:** In this real-world cohort of patients with HR+/HER2− metastatic breast cancer treated with CDK4/6 inhibitors, dichotomised BMI categories were not independently associated with survival outcomes. However, modelling BMI as a continuous variable revealed a non-linear (U-shaped) relationship, with increased risk at both the low and high ends of the BMI distribution. These findings suggest that the prognostic impact of BMI is non-linear and may be obscured by simple dichotomous categorisation.

## 1. Introduction

Breast cancer is the most frequently diagnosed malignancy among women and ranks as the second most common cancer globally. Approximately 70% of all breast cancers are hormone receptor-positive (HR+) and human epidermal growth factor receptor 2-negative (HER2-). Considering the high prevalence of HR+/HER2− disease, it remains clinically essential to identify modifiable prognostic factors, including those related to body composition [1].

Body mass index (BMI) has therefore attracted interest as a potential prognostic and predictive factor. Recent studies have investigated whether baseline adiposity measures, such as BMI and visceral fat, as well as weight changes during therapy, may affect treatment response or toxicity [2,3,4].

The prevalence of obesity has increased substantially over recent decades, and obesity has been associated with cancer progression through mechanisms including chronic low-grade inflammation and adipocyte-derived hormonal signalling, particularly in hormone-driven malignancies [3,5]. In hormone-driven breast cancer, oestrogens derived from adipose tissue may contribute to tumour proliferation and endocrine resistance [4].

However, the prognostic impact of obesity in the metastatic setting remains controversial. While some real-world cohorts have reported inferior clinical outcomes in patients with higher body mass index (BMI), others have demonstrated no significant association, highlighting ongoing uncertainty in this context [6,7,8].

Moreover, categorising BMI into broad dichotomous groups may oversimplify the biologically continuous and potentially non-linear relationship between adiposity and clinical outcomes. Such categorisation risks masking clinically relevant risk patterns at the lower and higher ends of the BMI spectrum, which may partly explain the conflicting findings across prior studies.

Interpretation of these findings is further complicated by heterogeneity in patient and treatment characteristics, study population sizes, body mass index (BMI) categorisation, and the clinical endpoints assessed. Moreover, in most studies conducted in advanced breast cancer, underweight patients were either excluded from the analyses or not classified as a distinct patient subgroup [9,10,11,12,13]. Additionally, it remains uncertain whether BMI influences not only tumour biology, but also the efficacy and toxicity profile of systemic therapies [14].

Recent preclinical studies have shown that CDK4 (cyclin-dependent kinase 4) and CDK6 may influence adiposity and metabolic regulation, suggesting that CDK4/6 inhibitors may also affect body fat distribution and skeletal muscle metabolism [15,16].

CDK4/6 inhibitors have become a standard component of therapy for HR+/HER2 negative metastatic breast cancer following the approval of palbociclib by the United States Food and Drug Administration (FDA) in 2015. Key clinical studies such as the PALOMA, MONALEESA, and MONARCH programmes have shown significant clinical benefits with CDK4/6 inhibitors like palbociclib, ribociclib, abemaciclib, and dalpiciclib (in certain regions) in this patient group [17,18,19,20].

Despite growing interest, studies evaluating the impact of BMI on the efficacy of CDK4/6 inhibitors have yielded inconsistent results, and the independent prognostic and predictive value of BMI in this setting remains unclear [20]. To address this knowledge gap, the present study aimed to investigate the association between baseline BMI and clinical outcomes, including progression-free survival, overall survival, and treatment-related toxicity, in patients with HR+/HER2-negative metastatic breast cancer treated with CDK4/6 inhibitors in combination with endocrine therapy in the metastatic setting.

## 2. Materials and Methods

### 2.1. Study Design and Patient Selection

This single-centre retrospective observational cohort study was conducted at a tertiary oncology centre. Medical records of patients who received CDK4/6 inhibitors (palbociclib or ribociclib) in combination with hormone therapy for HR+/HER2− metastatic breast cancer between January 2018 and May 2025 were reviewed, regardless of treatment line in the metastatic setting.

A total of 456 eligible patients were identified. Inclusion criteria were: (1) histopathologically confirmed HR-positive (oestrogen receptor [ER] and/or progesterone receptor [PR] expression ≥ 1%) and HER2-negative metastatic breast cancer; (2) receipt of palbociclib or ribociclib in the metastatic setting; and (3) availability of adequate clinical and radiological follow-up data. Patients younger than 18 years, male patients, patients without measurable target lesions according to RECIST version 1.1, and those with insufficient clinical and/or radiological follow-up (defined as fewer than two imaging assessments) were excluded. This criterion was applied to ensure reliable assessment of progression and response outcomes, and the same eligibility criteria were used for both PFS and OS analyses.

### 2.2. Data Collection and Evaluation

Demographic, clinical, and pathological data were extracted from electronic medical records, including age, menopausal status, Eastern Cooperative Oncology Group performance status (ECOG PS), histological subtype, tumour biomarkers (ER, PR, Ki-67), metastatic patterns (site and number), prior treatments, and treatment response. Endocrine resistance was defined according to the Advanced Breast Cancer 7 (ABC7) consensus criteria, classifying patients as endocrine-naive, primary resistance, secondary resistance, or endocrine insensitivity [21]. Baseline body mass index (BMI) was calculated at treatment initiation and categorised as <25 or ≥25 kg/m^2^ according to World Health Organisation criteria, consistent with prior CDK4/6 inhibitor studies. The number of underweight patients (BMI < 18.5 kg/m^2^) was very small, precluding a statistically reliable subgroup analysis; therefore, the standard WHO BMI subcategories were not used. However, this dichotomous categorisation was primarily used for comparability with prior studies and may not fully capture biologically meaningful, non-linear associations between adiposity and clinical outcomes. PFS was defined as the time from initiation of CDK4/6 inhibitor therapy to radiologically confirmed disease progression or death from any cause, whichever occurred first; patients without an event were censored at their last clinical or radiological assessment. Radiological assessments were performed approximately every 8–12 weeks in routine clinical practice, or earlier if clinically indicated, using standard cross-sectional imaging. Disease progression was determined from radiological reports documented in the medical records. OS was defined as the time from treatment initiation to death from any cause, with surviving patients censored at the date they were last known to be alive. Patients enrolled towards the end of the inclusion period had shorter follow-up and were right-censored at their last available clinical or radiological assessment if no event had occurred. Adverse events were evaluated and classified according to the National Cancer Institute Common Terminology Criteria for Adverse Events (CTCAE), version 5.0. Haematological and non-haematological AEs were documented separately. CDK4/6 inhibitors were initiated at standard fixed starting doses per product labelling recommendations and were not weight-adjusted. Dose reductions or delays were implemented at the treating physician’s discretion in accordance with standard toxicity management guidelines and were not predefined or modified based on BMI category. As this was a real-world retrospective cohort, treatment interruptions or discontinuations were permitted in line with routine clinical practice. Treatment adherence was assessed via medical record review, and all included patients had received at least one full treatment cycle and a subsequent response assessment. The primary endpoint was progression-free survival, while secondary endpoints included overall survival and treatment-related toxicity.

### 2.3. Statistical Analysis

All statistical analyses were conducted in R (version 4.4.2; R Foundation for Statistical Computing, Vienna, Austria), using the survival, survminer, and rstatix packages. Descriptive statistics were generated for all variables. The Shapiro–Wilk test was used to assess normality. Normally distributed variables were summarised as mean ± standard deviation, and non-normally distributed variables as median (minimum–maximum). Baseline characteristics were compared between BMI groups using the independent samples *t*-test or Wilcoxon rank-sum test for continuous variables, and the chi-square or Fisher’s exact test for categorical variables. To minimise confounding, propensity score matching (PSM) was performed using a 1:3 nearest-neighbour algorithm based on prespecified baseline covariates. Covariate balance before and after matching was assessed using standardised mean differences (SMDs), with values < 0.10 considered acceptable. The propensity score model included the following prespecified baseline covariates: age, ECOG performance status, oestrogen receptor status, progesterone receptor status, presence of visceral metastases, presence of bone metastases, and number of metastatic sites. Matching was performed using 1:3 nearest-neighbour matching without replacement, with a calliper width of 0.2 times the standard deviation of the logit of the propensity score. Due to limited overlap in propensity score distributions between BMI groups and the use of calliper-based matching, some patients could not be matched and were therefore excluded from the final matched cohort. Residual imbalance after matching was assessed using standardised mean differences, and, where appropriate, further adjusted for in multivariable regression models. Categorical covariates (e.g., oestrogen receptor, progesterone receptor, and HER2 status) were modelled as indicator variables in the propensity score model, and balance within each category was assessed separately using standardised mean differences and visualised in a Love plot. Survival curves for PFS and OS were estimated using the Kaplan–Meier method and compared with the log-rank test. Cox proportional hazards models were constructed to evaluate the independent association between BMI and survival outcomes. Variables with clinical relevance or significance in univariate analyses were included in multivariate models to ensure model parsimony and reduce the risk of overfitting. Model assumptions were verified using Schoenfeld residuals. Hazard ratios (HRs) with 95% confidence intervals (CIs) were reported. Statistical significance was defined as a two-sided *p*-value < 0.05. Missing data were handled using a complete-case approach for each analysis. No formal imputation was performed. The proportion of missingness for individual baseline covariates was low and, therefore, unlikely to materially influence propensity score estimation or multivariable models. For each analysis, the effective sample size and denominators are reported in the corresponding tables, which may therefore vary slightly across variables. Accordingly, only patients with complete data for all covariates included in the propensity score model were eligible for matching.

### 2.4. Ethical Considerations

The study was approved by the Institutional Ethics Committee of Kartal Dr Lütfi Kırdar City Hospital (Approval No: 2025/010.99/21/16) and conducted in accordance with the Declaration of Helsinki. Given the study’s retrospective design and the use of fully anonymised data, the ethics committee waived the requirement for written informed consent.

## 3. Results

**Figure 1:**
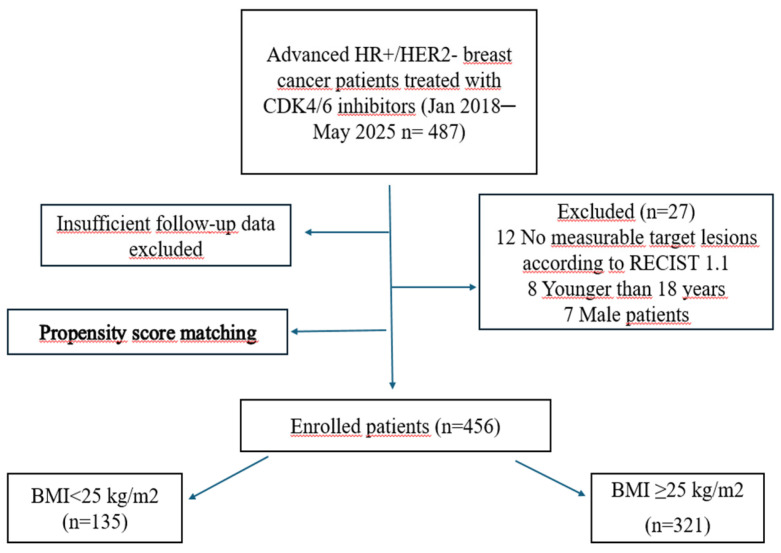
Flowchart of patient selection and categorisation based on body mass index (BMI).

A total of 456 patients with HR-positive/HER2-negative metastatic breast cancer treated with endocrine therapy plus a CDK4/6 inhibitor (ribociclib or palbociclib) were included in the analysis. At baseline, 321 patients (70.4%) had a BMI ≥ 25 kg/m^2^, and 135 (29.6%) had a BMI < 25 kg/m^2^. To reduce baseline imbalances between BMI groups, propensity score matching with a 1:3 nearest-neighbour approach was used, yielding a matched cohort of 220 patients (BMI ≥ 25: n = 154; BMI < 25: n = 66). The median age of the overall cohort was 56 years (range, 30–86). Patient selection and cohort derivation are shown in Figure 1. The matched cohort was subsequently used for all survival and regression analyses unless otherwise specified.

Baseline clinicopathological characteristics stratified by BMI are summarised for the unmatched and matched cohorts in Table 1. Before matching, BMI groups differed in menopausal status (*p* = 0.010) and stage at diagnosis (*p* = 0.014), whereas other baseline characteristics were broadly comparable (Table 1). These variables were subsequently evaluated as potential prognostic factors in Cox regression analyses of progression-free and overall survival.

**Figure 2:**
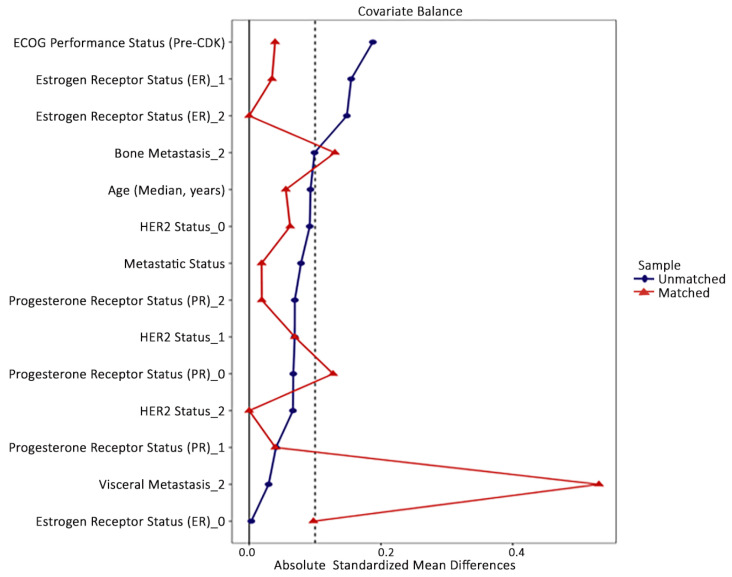
Love plot showing covariate balance between BMI < 25 kg/m^2^ and BMI ≥ 25 kg/m^2^ before and after propensity score matching, assessed using absolute standardised mean differences. The dashed vertical line indicates the threshold for acceptable covariate balance (standardised mean difference = 0.10).

After propensity score matching, overall covariate balance improved substantially, with most absolute standardised mean differences falling below 0.10 and all remaining values below 0.20, indicating acceptable residual imbalance (Table 1; Figure 2). Overall, propensity score matching resulted in improved baseline comparability between BMI groups.

**Figure 3:**
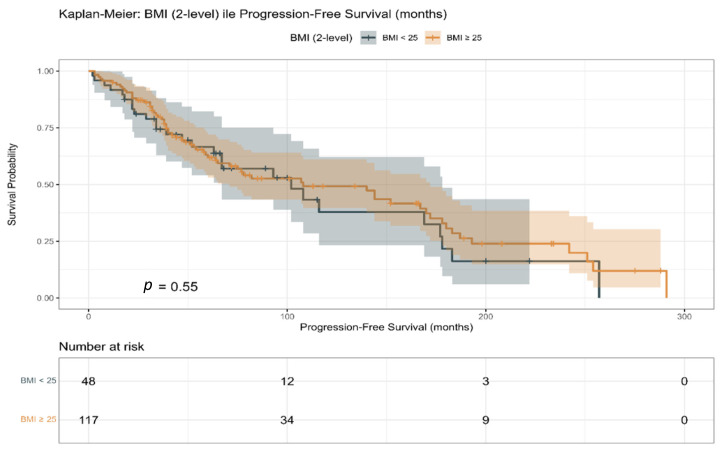
Kaplan–Meier curve for progression-free survival (PFS) stratified by body mass index (BMI) categories in the propensity score-matched study population.

In the propensity score-matched cohort, Kaplan–Meier analyses demonstrated no statistically significant difference in progression-free survival between patients with BMI < 25 kg/m^2^ and those with BMI ≥ 25 kg/m^2^ (log-rank *p* = 0.55; Figure 3). Similar results were observed in the unmatched cohort, with survival curves largely overlapping across BMI categories.

**Figure 4:**
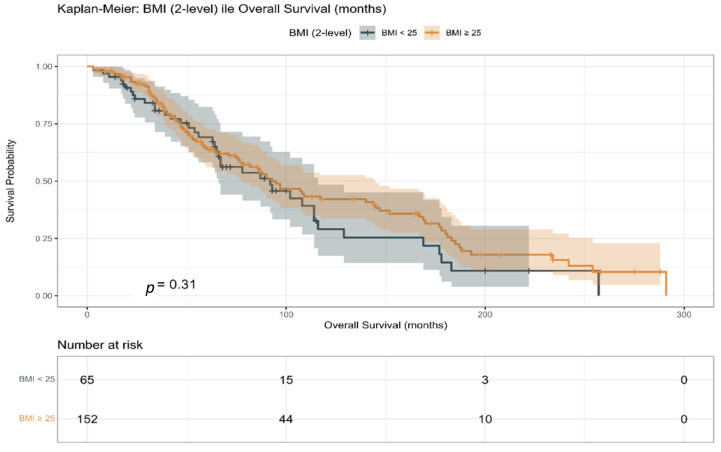
Kaplan–Meier curve for overall survival (OS) stratified by body mass index (BMI) categories in the propensity score-matched study population.

Overall survival did not differ significantly between BMI categories in the matched cohort (log-rank *p* = 0.31; Figure 4), consistent with the findings from the unmatched analysis. Visual inspection of the Kaplan–Meier curves showed substantial overlap, indicating no clinically meaningful difference in survival outcomes by dichotomised BMI. 

Given the absence of significant survival differences by dichotomised BMI in Kaplan–Meier analyses, multivariable Cox regression models were constructed to further explore independent prognostic factors and potential heterogeneity across BMI categories.

**Figure 5:**
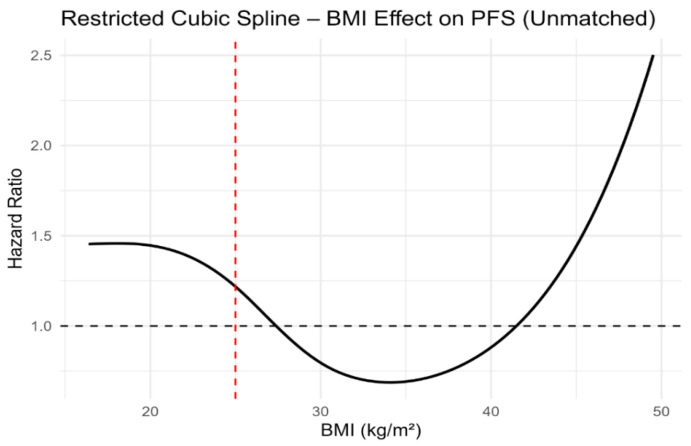
Restricted cubic spline analysis of body mass index and progression-free survival in the unmatched cohort. The red dashed vertical line indicates the BMI reference value of 25 kg/m^2^.

**Figure 6:**
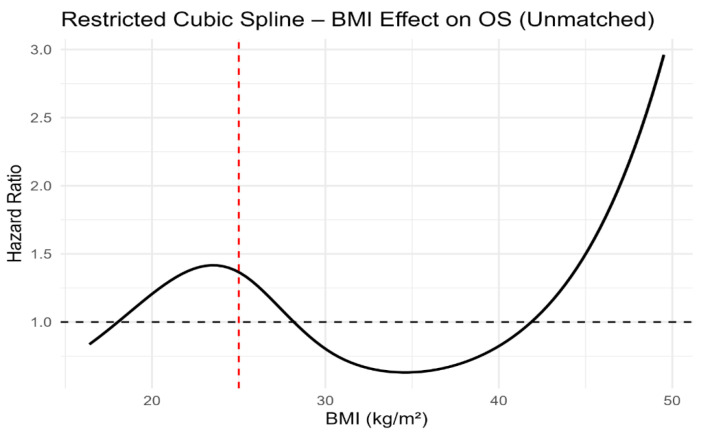
Restricted cubic spline analysis of body mass index and overall survival in the unmatched cohort. The red dashed vertical line indicates the BMI reference value of 25 kg/m^2^.

**Figure 7:**
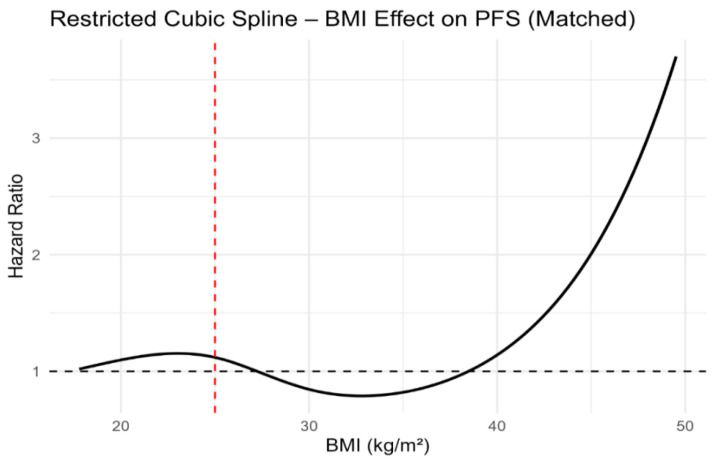
Restricted cubic spline analysis of body mass index and progression-free survival in the propensity score-matched study population. The red dashed vertical line indicates the BMI reference value of 25 kg/m^2^.

**Figure 8:**
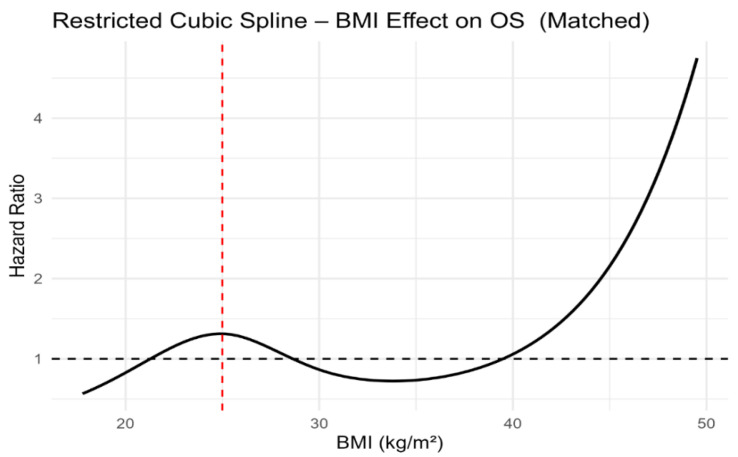
Restricted cubic spline analysis of body mass index and overall survival in the propensity score-matched study population. The red dashed vertical line indicates the BMI reference value of 25 kg/m^2^.

Although dichotomised BMI categories were not associated with PFS or OS, modelling BMI as a continuous variable using restricted cubic splines revealed a clear non-linear relationship with survival outcomes. In both unmatched and propensity score-matched cohorts, the lowest estimated hazard was observed at intermediate BMI values, with progressively higher risk towards the lower and higher extremes of BMI (Figure 5, Figure 6, Figure 7 and Figure 8). Importantly, this non-linear association was consistent across both unmatched and propensity score-matched cohorts. These findings suggest that dichotomous BMI categorisation may obscure clinically relevant risk patterns.

In univariable Cox regression analyses of PFS (Table 2), menopausal status, stage at diagnosis, liver metastasis, and metastatic burden were associated with PFS in the matched cohort. The magnitude and direction of these associations differed across BMI strata.

In multivariable Cox regression analyses of PFS (Table 3), stage at diagnosis remained the most consistent independent prognostic factor across the overall matched cohort and BMI strata. Although liver metastasis had a large effect estimate in the BMI < 25 kg/m^2^ subgroup, the corresponding confidence intervals were wide, indicating limited precision and potential for model overfitting, likely due to the sparse number of events in this stratum. These findings suggest heterogeneity rather than definitive subgroup-specific effects.

Univariable Cox regression analyses for OS are summarised in Table 4. Several clinicopathological factors, including menopausal status, stage at diagnosis, and metastatic burden, showed associations with OS in the matched cohort, with variability observed across BMI categories.

In multivariable Cox regression analyses for OS (Table 5), stage at diagnosis and menopausal status remained independently associated with OS in the overall matched cohort. In BMI-stratified models, specific covariates, including liver and bone-only metastasis, showed significant effect estimates in the BMI < 25 kg/m^2^ subgroup; however, these estimates were accompanied by wide confidence intervals, reflecting limited statistical power and model instability in this subgroup. Accordingly, these results should be interpreted as exploratory.

Overall, while BMI-stratified Cox regression analyses suggested potential heterogeneity in the prognostic impact of certain clinicopathological variables, the wide confidence intervals in the subgroup-specific models underscore the need for cautious interpretation.

Patients may have experienced more than one AE; therefore, categories are not mutually exclusive.

Across the cohort, rates of dose reductions, dose delays, and treatment discontinuation did not differ significantly by BMI category (all *p* > 0.05). Similar findings were observed in the matched cohort.

Haematologic adverse events were common, whereas non-haematologic adverse events were infrequent (Table 6). Neutropenia occurred at comparable rates across BMI groups; however, the distribution of severity differed in the overall cohort (*p* = 0.045). After matching, neither the incidence nor the severity distribution of neutropenia differed significantly by BMI (*p* = 0.561 and *p* = 0.336, respectively). Thrombocytopenia and anaemia were comparable across BMI categories in both overall and matched analyses (all *p* > 0.05). Non-haematologic adverse events (AST/ALT elevation, QTc prolongation, and creatinine elevation) were rare and did not differ significantly by BMI in either cohort (all *p* > 0.05). Overall, the safety profile of CDK4/6 inhibitors was broadly similar across BMI categories in both the overall and propensity score-matched analyses

## 4. Discussion

Body mass index (BMI) has long been recognised as a surrogate marker of nutritional and metabolic status and has been associated with heterogeneous prognostic effects across oncological populations. However, its clinical relevance in patients with hormone receptor-positive/HER2-negative metastatic breast cancer treated with modern targeted therapies remains uncertain [22,23]. In this context, our findings provide contemporary real-world evidence of the prognostic relevance of BMI in the era of CDK4/6 inhibitor-based therapy.

In this large, single-centre, real-world cohort, baseline BMI was not independently associated with progression-free survival (PFS) or overall survival (OS) among patients receiving CDK4/6 inhibitor-based therapy, a finding that persisted after propensity score matching. Although exploratory BMI-stratified Cox regression analyses suggested heterogeneity in the magnitude of certain covariate effects, the extreme hazard ratios and wide confidence intervals observed in some subgroup-specific models are most likely attributable to the small number of events and the resulting limited precision of the estimates. Accordingly, these subgroup-specific findings should be interpreted as exploratory and hypothesis-generating rather than definitive, and should not be overemphasised for clinical decision-making. The overlap of Kaplan–Meier survival curves in the propensity score-matched cohort is consistent with the absence of a clinically meaningful difference in survival outcomes between BMI categories. Although propensity score matching reduced the effective sample size, it substantially improved baseline comparability between BMI groups, prioritising bias reduction over maximal sample retention.

This finding aligns with recent real-world data examining the prognostic significance of BMI, especially in advanced-stage HR-positive/HER2-negative disease. A study involving a large cohort of 1456 patients from the Netherlands reported that, among patients receiving endocrine therapy ± CDK4/6 inhibitors, overweight and obese individuals did not experience significantly different overall survival (OS) or progression-free survival (PFS) compared to those of normal weight [8]. Unlike several prior studies, our analysis incorporated propensity score matching and non-linear modelling, thereby reducing confounding and allowing a more nuanced evaluation of BMI-related effects.

Similarly, in the analysis conducted by Franzoi et al., it was emphasised that BMI is not a clear and consistent negative prognostic indicator in metastatic disease, as it is in early-stage breast cancer [23].

Notably, in the adjuvant setting, the large randomised PALLAS trial evaluating palbociclib in combination with endocrine therapy did not demonstrate a survival benefit over endocrine therapy alone, despite a strong biological rationale for CDK4/6 inhibition. Although conducted in early-stage disease and not designed to evaluate BMI-related effects, these findings highlight that the prognostic and predictive relevance of patient-related factors may differ substantially between early- and metastatic-stage disease. This contrast further supports the need for refined, biology-based metrics beyond simple BMI categorisation when interpreting treatment outcomes in metastatic breast cancer [14].

Reports describing an “obesity paradox” in metastatic breast cancer should be interpreted cautiously, as such findings may reflect residual confounding, selection bias, or limitations of dichotomous BMI categorisation rather than an actual protective biological effect [24]. Our non-linear spline analyses further support this interpretation by showing increased risk at both the low and high extremes of BMI, rather than a monotonic protective effect.

Most importantly, restricted cubic spline analyses consistently demonstrated a non-linear association between continuous BMI and both PFS and OS across unmatched and propensity score-matched cohorts. These findings underscore the limitations of dichotomous BMI categorisation and support the use of continuous modelling approaches in prognostic research.

The increased estimated risk at very low BMI values may reflect frailty, sarcopenia, or cancer-related cachexia [25], whereas the higher risk observed at extreme obesity may be related to adverse metabolic and inflammatory states, altered drug pharmacokinetics, and obesity-associated endocrine dysfunction with activation of growth factor-mediated proliferative signalling pathways [26].

In recent years, it has been increasingly emphasised that the simple BMI value does not adequately reflect biological reality, and that the primary determinant is body composition (visceral adiposity, subcutaneous fat, skeletal muscle mass, sarcopenia, etc.). Kripa and colleagues have demonstrated that, in patients with metastatic HR+/HER2− disease undergoing CDK4/6 inhibitor therapy, sarcopenia and specific patterns of fat/muscle distribution are associated with survival [25].

Jung et al. also demonstrated that obesity defined by BMI in patients with metastatic breast cancer receiving a CDK4/6 inhibitor is associated with only a slight improvement in PFS. In contrast, the most determinative factor is obesity, defined by VAT on CT [1].

In three studies involving patients with HR+/HER2− metastatic breast cancer (MBC) treated with CDK4/6 inhibitors and endocrine therapy (ET), the VAT index was used instead of BMI, and better outcomes were reported in patients with high visceral adiposity (OR/HR: 0.44–0.48, *p* = 0.008–0.063). BMI is a simple measure calculated as body weight divided by height squared. In contrast, the VAT index evaluates visceral fat area measured on computed tomography (CT) slices at the level of the third lumbar vertebra, adjusted for height. The VAT index has shown a stronger correlation with progression-free survival (PFS) compared to BMI [26,27]. Lammers et al.’s large real-world cohort also supports this approach, as BMI alone is not an independent prognostic factor [8].

Our study’s neutral result regarding BMI is consistent with this increasingly substantial paradigm shift: in metastatic patients treated with CDK4/6 inhibitors, more refined indicators of body composition (visceral fat, muscle density, muscle mass, and sarcopenia) are increasingly important for clinical outcomes than broad BMI categories.

Indeed, Imbimbo et al. reported that changes in muscle and fat tissue over time in patients receiving a CDK4/6 inhibitor are associated with toxicity, dose reduction, and treatment discontinuation [28]. In this context, our study further supports the notion that BMI alone is not a robust prognostic marker, providing supplementary data that lays the groundwork for future body composition-based studies.

Another important finding of our study is that no significant difference was observed between palbociclib and ribociclib with respect to PFS or OS. This result is consistent with many studies supporting the ‘class effect’ of CDK4/6 inhibitors and indicating that all three agents have similar efficacy profiles in real-world data.

In Sreenath et al.’s Indian cohort, no significant difference was observed between palbociclib and ribociclib for PFS or OS; only a marginal benefit favouring ribociclib, approaching statistical significance, was observed [29].

In a multicentre series from Turkey, it has also been demonstrated that palbociclib and ribociclib have similar activity and tolerability profiles in real-world settings. Although some studies have reported a numerical or marginal superiority of ribociclib in progression-free survival (PFS), this difference has not been consistently observed across all analyses [30].

In our study, the lack of a significant difference between the two agents in both unmatched and matched analyses, consistent with the literature, indicates that, in clinical practice, factors such as appropriate patient selection, comorbidities, and access to treatment are more influential than agent choice.

From a toxicity perspective, the lack of significant differences in haematological and non-haematological adverse events across BMI groups indicates that body weight does not significantly alter the safety profile of CDK4/6 inhibitors. This finding is consistent with studies suggesting that various body composition parameters (e.g., sarcopenia, low muscle density) may be associated with haematological toxicity and dose reduction, but that coarse BMI classification is insufficient to capture these nuances.

Our study demonstrates that, in patients treated with CDK4/6 inhibitors, the toxicity profile is generally predictable and manageable, independent of BMI, consistent with the absence of a BMI effect observed in survival analyses. This suggests that more sophisticated tools are needed for toxicity prediction beyond BMI.

Overall, our findings contribute to the ongoing debate regarding the prognostic relevance of BMI in HR-positive/HER2-negative metastatic breast cancer treated with CDK4/6 inhibitors. Although BMI is an easily accessible clinical parameter, it may not adequately capture the complex biological and metabolic factors that influence survival and toxicity outcomes. Future prospective studies incorporating more precise assessments of body composition and metabolic health are warranted to clarify their prognostic and predictive roles. 

This study has certain limitations. Firstly, due to the study’s retrospective design, selection bias and measurement discrepancies may arise during patient selection. In addition, patients included near the end of the study period had relatively short follow-up, which may particularly limit the maturity of overall survival estimates. Although propensity score matching (PSM) effectively balances the groups, residual confounding from unmeasured or unrecorded variables cannot be ruled out. Secondly, BMI alone does not sufficiently reflect the biological complexity of body composition. Parameters such as visceral fat, subcutaneous fat distribution, skeletal muscle mass, and muscle density were not evaluated, thereby preventing more refined analyses of the impact of obesity and metabolic status on clinical outcomes. In addition, alternative BMI categorisations (e.g., quartiles or standard WHO subgroups) were not formally tested, which may have limited the assessment of potential threshold or non-linear effects associated with categorical cut-offs. Separate analyses of underweight patients were not feasible because of their very small numbers, which may have limited the ability to detect specific risks in this subgroup. Thirdly, changes in patients’ BMI over time were not considered; however, dynamic changes in weight and muscle mass during treatment have been shown to influence toxicity and survival. Additionally, the single-centre design may limit the generalisability of the findings. Finally, the inability to assess potential mediating mechanisms, such as inflammatory biomarkers, metabolic parameters, and pharmacokinetic data, limits our capacity to fully explain the biological effects of BMI. Moreover, institutional treatment patterns and patient selection criteria may differ from those at other centres or healthcare systems, further limiting the generalisability of the findings. Therefore, multicentre, prospective validation studies are essential before these findings can be widely applied to routine clinical practice.

Future research should focus not only on BMI but also on the detailed components of body composition, which are of great importance. In particular, prospective assessment of parameters such as visceral adiposity, subcutaneous fat, muscle mass, and muscle density using CT- or DEXA-based analyses will provide deeper biological insights into treatment response and toxicity profiles under CDK4/6 inhibitor therapy. Additionally, integrating biomarkers such as inflammatory markers, metabolic signatures, indicators of insulin resistance, and cytokine profiles can enhance understanding of the complex interactions between obesity and tumour biology. Given the increasing use of combination therapies with new targeted agents (e.g., PI3K/mTOR inhibitors), future studies should also investigate the prognostic and predictive value of BMI and body composition in these regimens. Multicentre, large-sample, prospective studies will play a critical role in validating findings and enhancing their generalisability to clinical practice.

## 5. Conclusions

Our study positions itself as a balancing factor among the conflicting results in the literature by demonstrating that BMI does not have an independent and substantial effect on survival and toxicity in patients with HR-positive/HER2-negative metastatic breast cancer receiving CDK4/6 inhibitor-based therapy. Collectively, our findings suggest that treatment decisions regarding CDK4/6 inhibitor therapy should not be guided by BMI categories alone, and that more refined measures of body composition are needed to optimise prognostic stratification.

## Figures and Tables

**Table 1:** Baseline clinicopathological characteristics by BMI in the unmatched and propensity score-matched cohorts.

	Unmatched			Matched		
Variable	BMI < 25 kg/m^2^ (n = 135)	BMI ≥ 25 kg/m^2^ (n = 321)	*p*-Value	BMI < 25 kg/m^2^ (n = 66)	BMI ≥ 25 kg/m^2^ (n = 154)	*p*-Value
Age (years)			0.094			>0.999
≤60	86 (63.7%)	175 (54.5%)		39 (59.1%)	92 (59.8%)	
>60	49 (36.3%)	145 (45.2%)		27 (40.9%)	62 (40.3%)	
Menopausal Status			0.010			0.333
Pre-/perimenopausal	48 (35.6%)	75 (23.4%)		23 (34.9%)	42 (27.3%)	
Postmenopausal	87 (64.4%)	246 (76.6%)		43 (65.2%)	112 (72.7%)	
ECOG PS			0.088			0.230
0	95 (70.4%)	196 (61.4%)		47 (71.2%)	95 (61.7%)	
≥1	40 (29.6%)	123(38.6%)		19 (28.8%)	59 (38.3%)	
Oestrogen receptor status (%)			0.352			0.126
1–10%	2 (1.5%)	1 (0.3%)		1 (1.52%)	0 (0.0%)	
10–50%	8 (6.1%)	22 (7.3%)		0 (0.0%)	5 (3.3%)	
>50%	121 (92.4%)	278 (92.4%)		65 (98.5%)	149 (96.8%)	
Progesterone receptor status (%)			0.201			0.218
Negative	10 (8.1%)	11 (3.9%)		5 (7.6%)	4 (2.6%)	
1–20%	35 (28.2%)	74 (26.7%)		15 (22.8%)	41 (26.6%)	
>20%	79 (63.7%)	192 (69.3%)		46 (69.7%)	109 (70.8%)	
Ki-67 status (%)			0.822			>0.999
<20	37 (32.2%)	75 (30.4%)		15 (26.8%)	35 (26.9%)	
≥20	78 (67.8%)	172 (69.6%)		41 (73.2%)	95 (73.1%)	
Stage at diagnosis			0.014			0.077
I-III	60 (44.4%)	184 (57.5%)		28 (42.4%)	87 (56.5%)	
IV	75 (55.6%)	136 (42.5%)		38 (57.6%)	67 (43.5%)	
Endocrine sensitivity status			0.940			0.293
Sensitive	102 (75.6%)	243 (76.4%)		44 (66.7%)	115 (74.7%)	
Resistant	33 (24.4%)	75 (23.6%)		22 (33.3%)	39 (25.3%)	
Visceral metastases			0.816			0.811
Yes	63 (52.9%)	154 (54.8%)		26 (39.4%)	65 (42.2%)	
No	56 (47.1%)	127 (45.2%)		40 (60.6%)	89 (57.8%)	
Bone metastases			0.300			0.489
Yes	110 (82.1%)	247 (77.2%)		58 (87.9%)	128 (83.1%)	
No	24 (17.9%)	73 (22.8%)		8 (12.1%)	26 (16.9%)	
Number of metastatic sites			0.410			0.371
1	26 (22.2%)	78 (28.2%)		16 (24.6%)	50 (33.1%)	
2	22 (18.8%)	54 (19.5%)		13 (20.0%)	32 (22.2%)	
≥3	69 (59.0%)	145 (52.4%)		36 (55.4%)	69 (45.7%)	
CDK4/6 inhibitor therapy			0.102			0.303
Ribociclib	100 (74.1%)	211 (65.7%)		37 (56.1%)	73 (47.4%)	
Palbociclib	35 (25.9%)	110 (34.3%)		29 (43.9%)	81 (52.6%)	
CDK4/6 inhibitor treatment lines			0.406			0.151
1	79 (60.3%)	193 (60.3%)		36 (54.6%)	93 (60.4%)	
2	25 (19.1%)	75 (23.4%)		14 (21.2%)	40 (26.0%)	
≥3	27 (20.6%)	52 (16.3%)		16 (24.2%)	21 (13.6%)	
A combination of CDK4/6 inhibitor therapy			0.324			0.086
Aromatase inhibitors	22 (52.4%)	62 (59.1%)		12 (50.0%)	37 (69.8%)	
Tamoxifen	11 (26.2%)	14 (13.3%)		8 (33.3%)	5 (9.4%)	
Fulvestrant	3 (7.1%)	9 (8.6%)		1 (4.2%)	2 (3.8%)	
Others	6 (14.3%)	20 (19.1%)		3 (12.5%)	9 (17.0%)	
Comorbidity status			0.069			0.836
Yes	36 (31.3%)	120 (41.7%)		19 (33.9%)	50 (36.8%)	
No	79 (68.7%)	168 (58.3%)		37 (66.1%)	86 (63.2%)	

Percentages are calculated using available data; denominators may vary due to missing values.

**Table 2:** Univariate Cox regression analysis of predictors for progression-free survival in the propensity score-matched cohort, stratified by BMI.

	ALL			BMI < 25			BMI ≥ 25		
Characteristics	HR	95% CI	*p*-Value	HR	95% CI	*p*-Value	HR	95% CI	*p*-Value
Age	0.99	0.97–1.01	0.181	1.0	0.97–1.04	0.943	0.98	0.95–1.0	0.088
ECOG	0.85	0.52–1.40	0.524	1.16	0.48–2.82	0.736	0.72	0.39–1.33	0.295
Menopausal status	0.56	0.35–0.90	0.016	0.32	0.11–0.88	0.028	0.66	0.38–1.16	0.152
Stage at diagnosis	4.78	2.71–8.30	<0.001	4.08	1.51–11.0	0.006	4.91	2.50–9.62	<0.001
Presence of liver metastasis	1.88	1.11–3.20	0.019	2.33	0.94–5.76	0.067	1.70	0.86–3.36	0.127
Presence of bone-only metastasis	0.97	0.60–1.57	0.898	1.53	0.63–3.68	0.346	0.80	0.45–1.44	0.462
Metastasis site number	1.31	1.05–1.63	0.016	1.32	0.88–1.98	0.180	1.31	1.01–1.70	0.043
Previous line chemotherapy	1.11	0.68–1.80	0.686	1.22	0.49–2.99	0.671	1.07	0.59–1.93	0.824
Presence of diabetes mellitus	1.13	0.56–2.28	0.741	1.44	0.47–4.39	0.525	0.97	0.38–2.46	0.949
Hypertension presence	0.97	0.54–1.75	0.929	1.45	0.51–4.16	0.488	0.81	0.39–1.66	0.562
CDK4/6 inhibitor treatment lines									
2	1.12	0.64–1.96	0.693	1.42	0.48–4.17	0.523	1.00	0.51–1.95	0.997
≥3	0.95	0.53–1.68	0.855	1.05	0.38–2.94	0.924	0.87	0.43–1.77	0.695

**Table 3:** Multivariate Cox regression analysis of predictors for progression-free survival in the propensity score-matched cohort, stratified by BMI.

	ALL			BMI < 25 kg/m^2^			BMI ≥ 25 kg/m^2^		
Characteristics	HR	95% CI	*p*-Value	HR	95% CI	*p*-Value	HR	95% CI	*p*-Value
Age	0.95	0.92–0.98	0.003	0.98	0.91–1.05	0.606	0.95	0.91–1.00	0.044
ECOG	0.99	0.52–1.88	0.977	1.14	0.30–4.41	0.845	0.99	0.42–2.30	0.976
Menopausal status	0.28	0.13–0.59	<0.001	0.22	0.04–1.18	0.078	0.32	0.12–0.86	0.024
Stage at diagnosis	4.92	2.39–10.1	<0.001	8.29	1.90–36.2	0.005	4.38	1.83–10.5	<0.001
Presence of liver metastasis	1.08	0.47–2.50	0.850	11.4	1.15–113	0.038	0.72	0.23–2.23	0.571
Presence of bone-only metastasis	0.67	0.29–1.57	0.356	1.43	0.20–10.1	0.718	0.64	0.21–1.96	0.432
Metastasis site number	1.71	1.14–2.58	0.010	1.16	0.57–2.35	0.675	2.08	1.18–3.65	0.011
Previous line chemotherapy	0.87	0.45–1.68	0.686	0.56	0.13–2.52	0.453	1.18	0.53–2.63	0.682
Presence of diabetes mellitus	0.76	0.33–1.74	0.516	2.10	0.33–13.2	0.430	0.68	0.23–2.01	0.484
Hypertension presence	1.43	0.69–2.94	0.338	0.72	0.15–3.34	0.674	1.78	0.69–4.58	0.234
CDK4/6 inhibitor treatment lines									
2	0.56	0.27–1.16	0.119	0.02	0.00–0.38	0.009	0.40	0.16–0.96	0.040
≥3	0.67	0.22–2.09	0.492	0.07	0.00–1.80	0.108	1.19	0.32–4.41	0.792

**Table 4:** Univariate Cox regression analysis of predictors for overall survival in the propensity score-matched cohort, stratified by BMI.

	ALL			BMI < 25 kg/m^2^			BMI ≥ 25 kg/m^2^		
Characteristics	HR	95% CI	*p*-Value	HR	95% CI	*p*-Value	HR	95% CI	*p*-Value
Age	1.00	0.98–1.02	0.874	1.03	1.00–1.06	0.090	0.98	0.96–1.01	0.156
ECOG	1.18	0.76–1.83	0.453	2.09	0.96–4.54	0.063	0.92	0.54–1.57	0.759
Menopausal status	0.50	0.33–0.77	0.002	0.23	0.09–0.59	0.002	0.65	0.39–1.08	0.097
Stage at diagnosis	3.01	1.85–4.89	<0.001	2.27	0.98–5.23	0.055	3.28	1.80–5.96	<0.001
Presence of liver metastasis	1.99	1.22–3.25	0.006	2.31	1.01–5.27	0.047	1.86	0.99–3.50	0.053
Presence of bone-only metastasis	1.07	0.69–1.66	0.778	1.94	0.88–4.25	0.099	0.84	0.49–1.44	0.527
Metastasis site number	1.24	1.04–1.49	0.019	1.21	0.83–1.76	0.315	1.25	1.02–1.54	0.034
Previous line chemotherapy	1.06	0.68–1.65	0.802	1.24	0.56–2.73	0.596	0.97	0.56–1.67	0.913
Presence of diabetes mellitus	1.31	0.71–2.42	0.393	1.59	0.59–4.29	0.362	1.14	0.51–2.53	0.746
Hypertension presence	1.13	0.68–1.88	0.632	2.10	0.85–5.22	0.108	0.89	0.48–1.65	0.706
CDK4/6 inhibitor treatment lines									
2	1.22	0.74–2.02	0.432	1.23	0.69–2.20	0.490	1.23	0.69–2.20	0.490
≥3	0.92	0.54–1.55	0.744	0.84	0.43–1.64	0.606	0.84	0.43–1.64	0.606

**Table 5:** Multivariate Cox regression analysis of predictors for overall survival in the propensity score-matched cohort, stratified by BMI.

	ALL			BMI < 25 kg/m^2^			BMI ≥ 25 kg/m^2^		
Characteristics	HR	95% CI	*p*-Value	HR	95% CI	*p*-Value	HR	95% CI	*p*-Value
Age	0.97	0.94–1.00	0.039	0.99	0.93–1.06	0.858	0.96	0.93–1.00	0.030
ECOG	1.14	0.65–2.02	0.643	2.83	0.85–9.42	0.089	0.96	0.46–2.01	0.917
Menopausal status	0.37	0.19–0.72	0.004	0.30	0.07–1.25	0.099	0.37	0.16–0.83	0.016
Stage at diagnosis	2.20	1.20–4.01	0.010	4.24	1.17–15.3	0.028	2.14	1.03–4.44	0.041
Presence of liver metastasis	1.58	0.79–3.14	0.196	14.4	2.49–83.5	0.003	1.04	0.41–2.62	0.931
Presence of bone-only metastasis	1.17	0.54–2.54	0.694	6.01	1.03–35.1	0.047	0.79	0.30–2.08	0.630
Metastasis site number	1.26	0.92–1.74	0.152	1.16	0.63–2.14	0.631	1.36	0.91–2.04	0.129
Previous line chemotherapy	0.95	0.54–1.68	0.862	0.86	0.26–2.87	0.801	0.98	0.49–1.97	0.954
Presence of diabetes mellitus	0.87	0.41–1.85	0.718	1.93	0.35–10.6	0.448	0.81	0.31–2.11	0.670
Hypertension presence	1.23	0.68–2.25	0.495	0.82	0.19–3.52	0.795	1.10	0.53–2.31	0.792
CDK4/6 inhibitor treatment lines									
2	0.75	0.39–1.44	0.388	1.02	0.96–1.08	0.472	0.92	0.43–1.98	0.838
≥3	0.47	0.20–1.12	0.089	2.55	0.62–10.5	0.194	0.62	0.23–1.70	0.354

**Table 6:** Treatment-related haematologic and non-haematologic adverse events by body mass index (BMI) categories in the overall cohort.

AEs	Total (N = 456)		BMI < 25 kg/m^2^ (n = 135)		BMI ≥ 25 kg/m^2^ (n = 321)	
	All Grade (n, %)	Grade 3/4 (n, %)	All Grade (n, %)	Grade 3/4 (n, %)	All Grade (n, %)	Grade 3/4 (n, %)
Haematologic AEs (overall)	423 (92.8)	191 (41.9)	129 (95.6)	65 (48.1)	294 (91.6)	126 (39.3)
Neutropenia	416 (91.2)	190 (41.4)	125 (92.6)	65 (48.1)	291 (90.7)	125 (38.9)
Thrombocytopenia	72 (15.8)	16 (3.5)	25 (18.5)	5 (3.7)	47 (14.6)	11 (3.4)
Anaemia	158 (34.6)	5 (1.1)	47 (34.8)	0 (0)	111 (34.6)	5 (1.6)
Non-haematologic AEs (overall)	56 (12.3)	10 (2.2)	17 (12.6)	3 (2.2)	39 (12.1)	7 (2.2)
ALT increased	34 (7.5)	10 (2.2)	12 (8.9)	3 (2.2)	22 (6.9)	7 (2.2)
AST increased	40 (8.8)	9 (2.0)	12 (8.9)	3 (2.2)	28 (8.7)	6 (1.9)
Creatinine increased	5 (1.1)	0 (0)	2 (1.5)	0 (0)	3 (0.9)	0 (0)
QTc prolongation	12 (2.6)	0 (0)	2 (1.5)	0 (0)	10 (3.1)	0 (0)

## Data Availability

The datasets generated and/or analysed during the current study are not publicly available but are available from the corresponding author on reasonable request.

## Abbreviations

AEs	adverse events
ALT	alanine aminotransferase
AST	aspartate aminotransferase
BMI	Body mass index
CDK	Cyclin-dependent kinase
CI	Confidence interval
CR	complete response
ECOG PS	Eastern Cooperative Oncology Group Performance Status
ER	Oestrogen receptor
ET	Endocrine therapy
HR	Hazard ratio
HER2	Human epidermal growth factor receptor 2
OS	Overall survival
PR	Progesterone receptor
PFS	Progression-free survival
PSM	Propensity score matching
RECIST	Response Evaluation Criteria in Solid Tumours
PR	partial response
PD	progressive disease
SD	stable disease
CT	computed tomography
DEXA	Dual-energy x-ray absorptiometry
